# Adult Consequences of Late Adolescent Alcohol Consumption: A Systematic Review of Cohort Studies

**DOI:** 10.1371/journal.pmed.1000413

**Published:** 2011-02-08

**Authors:** Jim McCambridge, John McAlaney, Richard Rowe

**Affiliations:** 1 Centre for Research on Drugs & Health Behaviour, Department of Public Health & Policy, London School of Hygiene & Tropical Medicine, London, United Kingdom; 2 Department of Psychology, University of Sheffield, Sheffield, United Kingdom; Simon Fraser University, Canada

## Abstract

**Background:**

Although important to public policy, there have been no rigorous evidence syntheses of the long-term consequences of late adolescent drinking.

**Methods and Findings:**

This systematic review summarises evidence from general population cohort studies of drinking between 15–19 years old and any subsequent outcomes aged 20 or greater, with at least 3 years of follow-up study. Fifty-four studies were included, of which 35 were assessed to be vulnerable to bias and/or confounding. The principal findings are: (1) There is consistent evidence that higher alcohol consumption in late adolescence continues into adulthood and is also associated with alcohol problems including dependence; (2) Although a number of studies suggest links to adult physical and mental health and social consequences, existing evidence is of insufficient quality to warrant causal inferences at this stage.

**Conclusions:**

There is an urgent need for high quality long-term prospective cohort studies in order to better understand the public health burden that is consequent on late adolescent drinking, both in relation to adult drinking and more broadly. Reducing drinking during late adolescence is likely to be important for preventing long-term adverse consequences as well as protecting against more immediate harms.

*Please see later in the article for the Editors' Summary*

## Abstract 2

In a systematic review of cohort studies of adolescent drinking and later outcomes, Jim McCambridge and colleagues show that although studies suggest links to worse adult physical and mental health and social consequences, existing evidence is of poor quality.

## Editors' Summary

**Background:**

The effects of alcohol intoxication (drunkenness), dependence (habitual, compulsive, and long-term drinking), and the associated biochemical changes, have wide-ranging health and social consequences, some of which can be lethal. Worldwide, alcohol causes 2.5 million deaths (3.8% of total) and 69.4 million (4.5% of total) of disability-adjusted life years (DALYs). Unintentional injuries alone account for about one-third of the 2.5 million deaths, whereas neuro-psychiatric conditions account for almost 40%. There is also a causal relationship between alcohol consumption and more than 60 types of disease and injury; worldwide, alcohol is estimated to cause about 20%–30% cases of esophageal cancer, liver cancer, cirrhosis of the liver, homicide, epilepsy, and motor vehicle crashes. There is increasing evidence that, in addition to the volume of alcohol consumed, the pattern of drinking has an effect on health outcomes, with binge drinking found to be particularly harmful. As the majority of people who binge drink are teenagers, this group may be particularly vulnerable to the damaging health effects of alcohol, leading to global concern about the drinking trends and patterns among young people.

**Why Was This Study Done?:**

Although there have been many published cohort studies reporting the longer term harms associated with adolescent drinking, the strength of this evidence remains unclear, which has implications for the objectives of interventions. For example, if adolescent drinking does not cause later difficulties, early intervention on, and management of, the acute consequences of alcohol consumption, such as antisocial behaviour and unintentional injuries, may be the most appropriate community safety and public health responses. However, if there is a causal relationship, there needs to be an additional approach that addresses the cumulative harmful effects of alcohol. The researchers conducted this systematic review of cohort studies, as this method provides the strongest observational study design to evaluate evidence of causality.

**What Did the Researchers Do and Find?:**

The researchers conducted a comprehensive literature review to identify relevant studies that met their inclusion criteria, which were: (1) data collection from at least two points in time, at least 3 years apart, from the same cohort; (2) data collection regarding alcohol consumption between the ages of 15 and 19 years old; and (3) inclusion of a report of at least one quantitative measure of effect, such as an odds ratio, between alcohol involvement and any later outcome assessed at age 20 or greater. The majority of these studies were multiple reports from ten cohorts and approximately half were from the US. The researchers then evaluated the strength of causal inference possible in these studies by assessing whether all possible contributing factors(confounders) had been taken into account, identifying studies that had follow-up rates of 80% or greater, and which had sample sizes of 1,000 participants or more.

Using these methods, the researchers found that, overall, there is consistent evidence that higher alcohol consumption in late adolescence continues into adulthood and is also associated with alcohol problems, including dependence. For example, one population-based cohort showed that late adolescent drinking can cause early death among men, mainly through car crashes and suicides, and there was a large evidence base supporting the ongoing impacts of late adolescent drinking on adult drinking behaviours—although most of these studies could not strongly support causal inferences because of their weak designs. The researchers also concluded that although a number of studies suggested links with late adolescent drinking to adult physical and mental health and social consequences, this evidence is generally of poor quality and insufficient to infer causality.

**What Do These Findings Mean?:**

The results of this study show that that the evidence-base on the long-term consequences of late adolescent drinking is not as extensive or compelling as it needs to be. The researchers stress that there is an urgent need for high quality long-term prospective cohort studies in order to better understand the public health burden associated with adolescent drinking in general and in relation to adult drinking. However, there is sufficient evidence to suggest that reducing drinking during late adolescence is likely to be important for preventing long-term adverse consequences as well as protecting against more immediate harmful consequences harms.

**Additional Information:**

Please access these Web sites via the online version of this summary at http://dx.doi.org/10.1371/journal.pmed.1000413.

- The World Health Organization has information about the global incidence of alcohol consumption
- The US-based Marin Institute has information about alcohol and young people
- The BBC also has a site on late adolescent drinking

## Introduction

Alcohol is responsible for approximately 4% of the global burden of disease [1]. This burden is higher in high income countries and among men, accounting for 11% of all male deaths in the World Health Organization (WHO) European region in 2004 [1]. There is global concern about drinking trends among young people, particularly in heavy episodic or “binge” drinking. Prominent among policy responses, in the UK and elsewhere, have been attempts to manage antisocial behaviour related to intoxication in public spaces [2]. Much less attention has been given to risks to adult health and well being.

There have been many cohort studies of the longer term harms associated with adolescent drinking. Some studies suggest that individuals “mature out” of late adolescent drinking patterns [3], whilst others identify enduring effects on drinking and broader health and social functioning in adulthood [4]. In the only available meta-analysis of life-course variability, Johnstone and colleagues [3] evaluated stability in drinking frequency and found settled patterns after the age of 30 following earlier marked discontinuity. There has, however, been no systematic review addressing the consequences of late adolescent drinking in adulthood.

If adolescent drinking does not cause later difficulties with which it may be associated, early intervention on and management of the acute consequences of alcohol consumption, such as antisocial behaviour and unintentional injuries [5], may be the most appropriate community safety and public health responses. If causal relationships do exist, however, this approach will not address the cumulative harms produced by alcohol, unless such intervention successfully modifies the long-term relationship with alcohol, which seems unlikely. The obstacles to causal inference are well known, and bias and confounding in particular must be addressed in cohort studies. A systematic review of cohort studies provides the strongest observational study design to evaluate evidence for causal inference [6]. We thus applied this approach to study the consequences of late adolescent drinking.

## Methods

Adolescent alcohol involvement and the potential for subsequent harm have been conceptualised and studied in many different ways. We sought therefore to evaluate the possible effects of any behavioural measure of adolescent alcohol consumption on any adult outcome. The durability of any observed effects is an important study theme due to the likely implications for public health.

### Search Strategy and Selection Criteria

**Figure 1:**
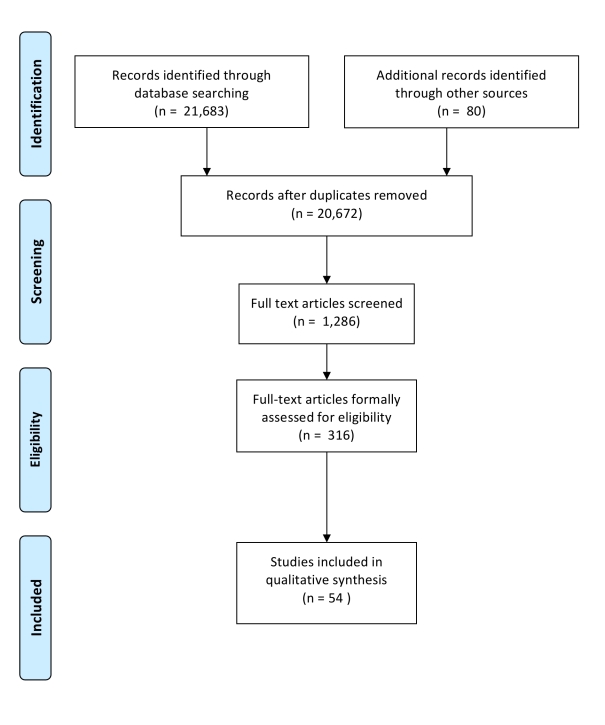
PRISMA Flowchart.

The data collection process is illustrated in Figure 1 and the PRISMA checklist is included as Text S1. The literature covering 1964 to 2008 inclusive was initially accessed via electronic databases as proposed by Egger and Davey Smith [7]. This start date identified the oldest cohort study included in the study by Johnstone and colleagues [3]. After piloting the following databases were searched: Medline (via both PubMed and MeSH); Web of Knowledge (including ISI Proceedings); Global Health Archive; Cinhal; PsychInfo; Embase; and HMIC. Configured for a PubMed search, the search terms were (1) Adolescen* OR teen* OR young person OR young people OR young adult; (2) Alcohol* OR binge drinking OR drinking culture OR problem drinking OR drinking problem* OR hazardous drinking OR substance [TI]; (3) Adult* [TI] OR cohort OR longitudinal OR prospective OR lifetime [TI]. Initial screening removed studies that were clearly unrelated to this review.

Citation searching used both backward and forward procedures, with the bibliographies of relevant studies checked and Science Citation Index used for subsequent citations of these papers. Three journals were hand searched: Addiction Abstracts; Addiction; and Journal of Studies on Alcohol and Drugs. The latter two have been published continuously during 1964 to 2008 and were selected following piloting. A data collection protocol was developed and the entire process was undertaken twice, on the second occasion by a research assistant blinded to the outcome of the first. All subsequent study tasks were also duplicated. Only peer-reviewed published data were used and further unpublished information was not sought from authors. Finally, experts, including the authors of included primary studies, were contacted to identify additional studies that had been missed.

The following selection criteria were applied independently by two researchers. Studies of drinking behaviour were included if they collected data on at least two points in time, were at least 3 y apart, and from the same cohort. Data collection regarding alcohol consumption was required between the ages of 15 and 19 y old (or between 9th grade at school or first year of university if age not specified). Drinking is normative in this age group and approaches peak levels towards the end of this age range and into the early 20s in most high income countries [8]. Studies were also required to include a report of at least one quantitative measure of effect, such as an odds ratio (OR), between alcohol involvement and any later outcome assessed at age 20 or greater. Cohorts formed from general population sources, including college students and military conscripts, were included. Studies based on selected or special populations such as children of alcoholics, mental health patients, and offenders were excluded.

### Data Analysis

Quality appraisal of included studies was undertaken to evaluate the potential for bias and the adequacy of control for confounding. We gave particular attention to socioeconomic deprivation and other early life sources of vulnerability, as well as indications of other adolescent behavioural problems, in assessing confounding. We designated studies as having stronger capacity for casual inference in relation to the aims of this review if residual confounding in these areas was assessed as unlikely to be important by two reviewers and they had at least one of the following characteristics: (1) follow-up rates of 80% or greater; or (2) sample sizes of 1,000 participants or more. These characteristics identify two forms of bias at the individual study (attrition bias) and at the review level (small studies having disproportionate influence in reviews), respectively. Also if a study had both these characteristics, it was deemed to have stronger capacity for causal inference if both reviewers agreed there was reasonable control of confounding even though residual confounding was nevertheless still likely. We considered that adjustment for some factors while leaving major individual psychosocial confounders uncontrolled, did not constitute reasonable control of confounding. This subset of studies with stronger capacity for causal inference is individually discussed in the narrative presentation of results in order to summarise the evidence base. Two researchers agreed on all bar two studies (Kappa 0.91) [9],[10], for which disagreements about the strength of control for confounding were resolved by discussion. Meta-analysis of pooled outcomes from these observational studies was deemed inappropriate only after consideration of the nature of findings from included studies, because of the potentially misleading nature of such summary effect estimates in the context of uncontrolled bias and confounding [7].

## Results

The majority of included studies (*n = *35) were multiple reports from ten cohorts (see Table 1), with the Swedish Conscript Study (SCS) of male conscripts providing nine separate reports [11]–[19]. The remainder of included studies (*n = *19) originated from separate cohorts (see Table 2). Tables 1 and 2 present selected study characteristics including variables involved in eligible measures of effect. Altogether a total of 54 studies were eligible for inclusion in this review [9]–[62]. This literature has grown rapidly in recent years, with approximately two-thirds of studies (*n = *35) published since 2001. Approximately half of all reports (*n = *26) were from US studies, ten were from Sweden, eight from Britain, four from New Zealand, three from Australia, two from Finland, and one from the Netherlands. More than half (*n = *30) originated from school-based cohorts. Birth cohorts were more likely to be the subject of multiple studies (*n = *11/14). Nineteen (35%) studies, based on eight different cohorts, were assessed as having stronger capacity for causal inference (see Tables 1 and 2), and we focus primarily on these studies. The presentation of main results is organised by principal outcomes evaluated, with quantitative data presented only from the subset of 19 studies.

**Table 1:** Multiple reports for ten cohort studies.

Study	Cohort Type	Author Name	Year	Age T1 (y)	Age T2 (y)	Final Sample Size (*n*)	Follow-up Rate (%)	T1 Adolescent Behavioural Variable(s)	T2 Adult Outcomes	Stronger Capacity for Causal Inference
**British Cohort Study**	National birth cohort	Viner	2007	16	30	4,911	81	Binge drinking; frequent regular drinking	Social class; AD (CAGE); weekly AU above recommended limits; illicit drug use; heavy smoking; psychological morbidity; mental health problems; homelessness; conviction; education; pregnancy, significant accidents	No
		Cable	2008	16	30	13,919	Not clear	AU frequency and quantity - in typologies	AD (CAGE)	No
**CHDS**	Local birth cohort	Wells	2004	16	21	859	90	AU frequency and quantity, problems (modified Rutgers Alcohol Problem Index) to form a latent class	AU, AA, AD; nicotine and illicit drug dependence; mental health outcomes; education and employment; sexual relationships; offending	Yes
		Wells	2006	18	21	983	96	AUD (AA and AD)	AUD (AA and AD)	Yes
**Dunedin Multidisciplinary Health and Development Study**	Local birth cohort	Casswell	1997	18	21	770	84	AU frequency and quantity	AU frequency and quantity, related problems (latent variable)	No
		McGee	2000	15, 18	21	871	Not clear	AU at both ages	Mental disorder; cannabis use	No
**MFCS**	National school cohort	Schulenberg	1996	18	23/24	6,862	63	Binge drinking	Binge drinking	No
		Merline	2004	18	35	10,225	61	Heavy drinking	Heavy drinking	No
		Merline	2008	18	22; 26; 35	21,137^a^	80	AU frequency and heavy drinking	AU frequency and heavy drinking: AA and AD (DSM) (measured only at age 35)	Yes
**National Child Development Study**	National birth cohort	Ghodsian	1987	16	23	9,337	79	AU frequency and quantity	AU quantity and frequency	No
		Power	1990	16	23	12,311	76	AU frequency and quantity	AU frequency and quantity; employment variables	No
		Jefferis	2005	16	23; 33; 42	9,527, 8,620, 8,600	79, 72, 72	Binge drinking	Binge drinking	No
		Maggs	2008	16	23	Not clear	Not clear	AU weekly quantity	AU weekly quantity	Yes
		Staff	2008	16	42	9,107	76	AU weekly quantity (categorised)	Educational attainment	Yes
**RAND Adolescent/Young Adult Panel (Project ALERT)**	School cohort	Tucker	2003	18	23	1,534^b^	71	AU frequency on 0–11 scale	Regular smoking	No
		D'Amico	2005	18	23; 29	1,986	30	AU as above	AUD (DSM)	No
		Bogart	2005	18	29	1,138^c^	Not clear	AU as above	AU quantity and frequency; negative consequences; heavy episodic drinking	Yes
		Bogart	2007	18	29	2,376	Not clear	AU as above	Life satisfaction	Yes
		Collins	2007	18	29	454^d^	Not clear	AU as above	Marital status (divorce)	No
**Seattle Social Development Project**	School cohort	Guo	2001	16	21	Approx. 759	Approx. 94	AU any in past month	AUD and AD (DSM)	No
		Oesterle	2008	15–18	21; 24; 27	773	96	Heavy episodic drinking	Positive functioning; AUD (DSM)	No
**SCS**	National conscription for military service	Andreasson	1988	18–20	33–35	49,464	99	AU weekly quantity (categorised)	Mortality	Yes
		Andreasson	1990	18–20	33–35	8,226^e^	95–99	AU as above	Hospitalisation for accidents, or gastro-intestinal, respiratory, musculo-skeletal, or infectious disorders	Yes
		Andreasson	1991a	18–20	38–40	49,464	99	AU as above	Mortality	Yes
		Andreasson	1991b	18–20	33–35	49,464	99	AU as above	Psychiatric hospital admission	Yes
		Andreasson	1993	18–20	33–35	49,464	99	AU as above	Hospital admission for alcoholism	Yes
		Stattin	1995	18–20	33–35	7,577^e^	99	Composite problems measure (incl. AU, hangover, intoxication, relief drinking, arrested drunk)	Criminal convictions, mortality	Yes
		Karlsson	1997	18–20	25–27	8,122^e^	99	AU quantity, intoxication and hangover regularity, relief drinking	Drink driving; public drunkenness	Yes
		Romelsjo	1999	18–20	43–45	49,618	99	AU daily quantity (categorised)	Mortality, myocardial infarction, stroke	Yes
		Stenbacka	2003	18–20	45–47	7,577^e^	93	Composite problems measure (incl. AU, hangover, intoxication, relief drinking, arrested drunk)	Hospitalisation or mortality for alcohol or drugs (separately)	Yes
**UCLA Longitudinal Study of Growth and Development**	School cohort	Locke	2001	18	26; 35	426	Not clear	Latent alcohol involvement factor (AU quantity and frequency, drinking at school/work, intoxication)	Latent alcohol involvement factor (as in adolescence except AUD replacing intoxication measure); latent dysphoria factor	No
		Locke	2003	18	26; 35	305^f^	Not clear	Latent alcohol involvement factor (as above)	Latent alcohol involvement and dysphoria factors as above at age 26, age 35 marital satisfaction, perceived opportunity, divorce, relationship and job satisfaction, job stability	No
		Locke	2004	18	26; 35	305f	Not clear	Latent alcohol involvement factor (as above)	Latent alcohol involvement and dysphoria factors as above	No
**Victorian Adolescent Health Cohort**	School cohort	Bonomo	2004	15–17	20/21	1,601	82	AU frequency and binge drinking	AD	No
		Patton	2007	15–17	24	1,520	78	AU quantity (categorised)	AU, weekly/daily cannabis use concurrent high risk alcohol/cannabis use, education, employment, relationships, parenthood, tobacco smoking, other drug use and problems measures	No

AA, alcohol abuse; AD, alcohol dependence; AU, alcohol use; AUD, alcohol use disorder; Beh, behaviour.

a Selected those who completed first postschool follow-up.

b Nonweekly smokers only.

c Women not married at age 18 only.

d Married by age 23 only.

e Stockholm County only.

f Women only.

**Table 2:** Individual cohort reports.

Study	Cohort Type	Author Name	Year	T1 Age (y)	T2 Age (y)	Final Sample Size (*n*)	Follow-up Rate (%)	T1 Adolescent Behavioural Variable(s)	T2 Adult Outcomes	Stronger Capacity for Causal Inference
**AHRS**	Community cohort	Jackson	2002	13–20 (mean 16.7)	18–25	1,814	88	AU frequency and heavy drinking	Au frequency and heavy drinking; regular and heavy tobacco smoking initiation and cessation	Yes
**Alcohol Misuse Prevention Study**	School cohort	Bingham	2005	17–18	23–24	1,987^a^	Not clear	AU quantity and frequency, drunkenness, binge drinking, drinking consequences	AU quantity and frequency, drunkenness, bingeing, AU disorders, drinking consequences	No
**Amsterdam Growth and Health Longitudinal Study**	School cohort	Koppes	2000	16	21	150	52	Total and beverage specific AU quantity	Total and beverage specific AU quantity	No
**Boston 13 year Longitudinal Project**	School cohort	Stein	1993	15– 18	26	785	79	AU frequency	AU quantity and frequency. Cannabis and other drug use. Work-related variables	No
**Cambridge Study of Delinquent Development**	School cohort	Shepherd	2004	16– 18	32	378^b^	94	Heavy weekly drinking	Illness and injuries	No
**FinnTwin 16– 25 Study**	National birth cohort	Viken	2007	18	25	3,028^c^	92	Alcohol problems (Rutgers Alcohol Problem Index)	Alcohol problems (Rutgers Alcohol Problem Index)	No
**Health in Transition Study**	School cohort	Toumbourou	2004	17– 19	21	1,596	48	AU quantity (within recommended limits)	AU quantity (within recommended limits), alcohol related harms	No
**Michigan Study of Adolescent Life Transitions**	School cohort	Peck	2008	18	21; 28	578	67	AU	AU frequency and heavy drinking (intoxication)	No
**Minnesota Longitudinal Study of Parents and Children**	Local birth cohort	Englund	2008	16	23; 26; 28	178^d^	Not clear	AU quantity	AU (quantity at 23, 26); AUD (DSM) at 28	No
**National Education Longitudinal Study**	National school cohort	Chatterji	2006	15– 16; 17– 18	26	7,604^e^	Not clear	AU any past month; heavy drinking	Graduated high school on time, diploma achievement, college entry, college graduation	No
**NYLS**	School cohort	Kandel	1986	15– 16	24– 25	1,004	83	Lifetime drinking ten times or more	Frequency of tobacco, alcohol, illicit drug use, prescription drug use, employment and family role measures, education, delinquency, physical and mental health	Yes
**North Karelia Youth Project**	School cohort	Paavola	2004	15	21; 28	657; 640	73; 71	AU frequency	AU, smoking, physical activity (all frequencies)	No
**Oregon Adolescent Depression Project**	School cohort	Rohde	2001	15– 19	24	940^f^	85	AUD diagnosed, symptoms only or nonproblematic	AUD, substance use disorder, depression, anxiety (all DSM), daily smoking, borderline and antisocial personality disorder symptoms	No
**Project Family**	School cohort	Mason	2008	16; 18	21– 22	313	Not clear	AU frequency and quantity; heavy drinking	Major depressive disorder (DSM)	No
**Young Adult Follow-up Study**	School cohort	Donovan	1983	15– 16	21– 22	403^g^	93	AU beverage specific and overall quantities, being drunk, negative consequences; years as a problem drinker	Problem drinker (intoxication frequency and negative consequences)	No
**Youth Development Study**	School cohort	McMorris	2000	17– 18	22	780	78	AU frequency	AU frequency; work hours	No
**Unnamed**	Local birth cohort	Wennberg	2000	18	25; 36	212	Not clear	AU quantity	Frequency of intoxication at 25, AU quantity at 36	No
	School cohort	Shope	2001	15– 16	23– 24	4,403^h^	100	Alcohol use/misuse (four categories)	Serious motoring offences; serious car crashes	Yes
	School cohort	Repetto	2004	14– 15	20– 21	458^i^	67	AU quantity	Depressive symptoms	No

AA, alcohol abuse; AD, alcohol dependence**;** AHRS, Adolescent Health Risk Study**;** AU, alcohol use; AUD, alcohol use disorder.

a Includes only not married/cohabiting young adults and sampled only those with a driving licence. Also partial overlap between this sample and Shope.

b Men only.

c Same-sex twins only.

d Low income first born sample.

e Various earlier data requirements met.

f AUD at 18 excluded in analyses examining the course of AUD.

g Previously participated in all four survey waves.

h Excluded those not living with either parent and those already driving at study entry, and those who did not obtain a driving license during the study period.

i Black only.

### Mortality

The risk of premature death associated with late adolescent drinking has been evaluated only in the SCS, after 15, 20, and 25 y [12],[15],[17]. When the male study population was approximately aged 34, late adolescent heavier drinkers (>250 g per week) were twice as likely (OR = 2.1, 95% confidence interval [1.4–3.2]) to have died compared to moderate drinkers (<100 g per week) [12]. This effect was attenuated by age 39 y (OR = 1.46 [1.05–2.04]) at which time potential confounding was more tightly controlled [15]. The majority of deaths at both study intervals were caused by car crashes and suicides. Car crashes were the leading cause of death at younger ages, after which time suicides predominated (see below) [15].

The risk of death among heavier adolescent drinkers due to alcohol-specific causes (International Classification of Diseases 8 [ICD 8] codes 291, 303, and 980 for alcohol psychosis, alcoholism, and alcohol intoxication, respectively) was high (OR = 13.7 [5.3–35.5]) compared to moderate drinkers, as were deaths due to liver cirrhosis and pancreatitis (ICD8 codes 571 and 577; OR = 11.0 [3.2–45.1]), though there were few such cases [15]. Heavier drinkers also differed from others in the ways in which other psychosocial factors impacted upon mortality risk. Among all conscripts high psychosocial risk (defined as 5 or more risk factors, compared to 0–2 factors) was associated with a 3-fold elevation in observed mortality (OR = 3.0 [2.3–4.0]). Among heavier drinkers there was no independent effect of psychosocial risk factor groups on mortality (OR = 1.3 [0.7–2.7]), meaning that “good social adjustment as indicated by absence of other risk factors constitutes little or no protection from an increased risk of premature mortality among high consumers of alcohol” [15].

Alcohol consumption was categorised slightly differently in the 25-y follow-up, complicating direct comparisons with earlier data [17]. Those drinking 15 g per day or greater were at heightened risk of early mortality (OR = 1.37 [1.01–1.85]) in comparison with a reference group of abstainers, with the risk slightly more pronounced among those drinking 30 g per day or more (OR = 1.53 [1.08–2.16]). Alcohol was estimated to have caused 14% of all deaths. Neither trends in protective effects on myocardial infarction hospitalisation or death nor on risk of stroke approached statistical significance [17].

### Alcohol Consumption

More than 20 studies provided evidence of associations between late adolescent alcohol consumption and subsequent drinking in adulthood, with one study reporting no associations based on a limited measure of alcohol involvement (see Tables 1 and 2 and below) [33]. There were five studies with stronger capacity for causal inference, four of which were published since 2004.

In the New Zealand birth cohort Christchurch Health and Development Study (CHDS), effects of an age 16 latent class variable on all drinking frequency and quantity outcomes at age 21 survived extensive adjustment for covariates. These controls eliminated apparent relationships between age 16 drinking and most other outcomes [60]. Latent classes were formed by consumption and frequency measures and were most importantly influenced by the largest amount consumed on a single occasion in the past 3 mo. In the British birth cohort National Child Development Study (NCDS) effects of overall weekly consumption at age 16 on this same measure were observed until age 23 y, as far as was studied [38].

Kandel and colleagues [33] found no direct relationship between having drunk alcohol ten times ever by age 16 and alcohol consumption behaviours at age 24–25 y in the New York Longitudinal Study (NYLS) cohort representative of school attenders in that state. Bogart and colleagues [9] found a small effect of drinking frequency measured using a 12-point scale at age 18 on whether any alcohol was being drunk (OR = 1.08 [1.01–1.15]) and on heavy episodic drinking (OR = 1.11 [1.01–1.22]), with no effect on overall monthly consumption among 29-y-old women in a subgroup analysis of the RAND cohort.

Both monthly frequency of consumption and heavy episodic drinking at age 18 were related to the same measures at 22, 26, and 35 y in the nationally representative US Monitoring the Future Cohort Study (MFCS) [43]. The MFCS identified these effects to be much larger than all other adolescent sociodemographic, parental, psychological, and behavioural predictors of both drinking frequency and heavy drinking outcomes. Standardised regression coefficients for both variables were on average at least twice as large as any other predictor across all follow-up intervals. These effects diminished over time, for example being 0.34, 0.21, and 0.18 for drinking frequency at the three ages (all *p<*0.001). They were also stronger for males than females in heavy drinking (regression coefficients approximately three times larger than any other), whereas there was no evidence of gender difference in monthly consumption frequency [43].

### Alcohol Problems Including Dependence

All studies assessing alcohol problems or dependence in adulthood found statistically significant associations with late adolescent drinking. Among seven studies with stronger capacity for causal inference, three were drawn from the SCS and two from the CHDS cohorts.

In CHDS, effects of age 16 alcohol consumption latent class on *Diagnostic and Statistical Manual of Mental Disorders, Fourth Edition* (DSM-IV) alcohol dependence at age 21, were larger than on any other alcohol-related outcome [60]. Diagnosis of alcohol abuse at age 18 was related to later alcohol diagnoses at 21 y (abuse OR = 2.6 [1.7–4.1], dependence OR = 3.0 [1.2–7.2]) [61]. Diagnosis of dependence at age 18 was associated with age 21 diagnosis of abuse at 21 (OR = 3.5 [1.8–6.7]) and more strongly with dependence (OR = 15.5 [6.0–40.1]) after good control for confounding in this birth cohort [61].

Similar to the findings on alcohol consumption, the RAND female study [9] found a small effect of age 18 drinking frequency measured on a 12-point scale on negative consequences attributed to alcohol in the past year at age 29 (OR = 1.12 [1.04–1.21]). There was no effect on the number of these consequences. As with their previous findings, these effects were smaller than the protective effects of marriage on women's drinking behaviour at 29. Longer term consequences to age 35 were again apparent in MFCS, with heavy episodic drinking at 18 being predictive of both DSM-IV abuse and dependence (standardised regression coefficients 0.09 [*p<*0.01] and 0.08 [*p<*0.001], respectively). Unlike effects on alcohol consumption, however, the effects of adolescent heavy drinking on alcohol problems at 35 were no longer larger than other possible component causes including parental drinking, theft/property damage, marijuana, and other illicit drug use at age 18 (regression coefficients broadly similar to heavy drinking and statistically significant) [43].

At approximately age 34, heavy drinking (>250 g per week) young men at age 19 were 2.3 (1.8–2.9) times more likely to have been hospitalised for alcoholism than low risk drinkers (<100 g per week) in the SCS [16]. Elevated risk also in the intermediate category provided evidence of a dose response relationship (OR = 1.6 [1.3–1.9]). Among other predictors, the relative risk was greater only among those having had earlier contact with police or child care authorities [16]. In the Stockholm Country subset of the SCS heavier drinking at conscription was not associated with public drunkenness offences 7–8 y later (approximate age 26–27 y) after adjustment for confounders [19]. After 26–27 y of follow-up of the national cohort (approximate age 45–46 y), a summary measure of problematic drinking at conscription was not associated with hospitalization or mortality with an alcohol diagnosis (OR = 1.33 [0.89–1.99] in the subgroup who had not used cannabis) [18].

### Car Crashes and Drink Driving Offences

SCS mortality data have already been presented. Among Stockholm County conscripts, heavier alcohol consumption was not associated with drinking and driving offences after 7–8 y with adjustment for confounders [19]. The relative risk of car crash fatality in the entire SCS, however, was 2.3 (1.2–4.3) for heavier drinkers (>250 g per week) compared to moderate drinkers and 8.0 (2.2–28.9) compared to abstainers after 20 y of follow-up [15]. Moderate drinkers (<100 g per week), were also at elevated risk compared to abstainers (OR 3.5 [1.1–10.7]). CHDS identified a possible effect on any drink driving offences by age 21 of borderline statistical significance attributable to age 16 drinking pattern and no effect on speeding [60]. Shope and colleagues [53] found that the possible effects of a combined measure of alcohol use and problems assessed at age 15 before driving began on the numbers of serious crashes and driving offences to ages 23–24 were not robust to confounding in a US school cohort.

### Other Criminal Convictions

The CHDS found no effect of age 16 drinking latent class on court convictions or property offences by age 21 [60]. Effects on numbers of violent offences were, however, identified to be robust to adjustment for background variables. In the Stockholm County segment of the SCS a dichotomised adolescent alcohol problems measure was predictive of having any officially recorded criminal convictions during 15 y of follow-up (OR = 1.31, confidence interval not provided, *p<*0.001) [11].

### Mental Health

There were no age 16 alcohol effects on any of the mental health outcomes (major depression, anxiety disorder, suicidal ideation, and suicide attempt, all *p>*0.5 after adjustment) at age 21 assessed in CHDS [60]. There were no associations between age 15–16 drinking lifetime prevalence and having seen a mental health professional, nor on depressed mood at age 24–25 in the NYLS [33].

As noted above, suicide was the leading cause of death over the 20 y of the SCS mortality studies, with heavier drinkers at greater risk than moderate drinkers (OR = 1.7 [1.0–2.8]) and abstainers [15]. Other risk factors were more strongly associated with suicide, including number of friends (having none compared to having more than 3, OR = 3.1 [1.5–6.2]). Although there was also a greater risk of psychiatric hospitalisation after 15 y in the SCS (OR 1.8 [1.5–2.1]), the nonaddiction mental health consequences are difficult to appreciate as approximately two-thirds of all admissions involved alcoholism or drug addiction [14].

### Tobacco Smoking

An observed association between age 16 drinking patterns and DSM-IV nicotine dependence at age 21 disappeared after adjustment for covariates in CHDS [60]. In the US Adolescent Health Risk Study (AHRS) [31] small effects of occasional and heavy drinking on smoking initiation and cessation were identified over a 5-y interval into the early 20s. ORs for both regular smoking and half pack a day smoking suggested small effects, with lower confidence intervals near 1. These were statistically significant for all initiation analyses (among nonsmokers) but not in all cessation analyses (among baseline smokers). No effects of having drunk alcohol 10 times or more by age 15–16 on lifetime smoking prevalence by 24–25 y were observed in the NYLS [33].

### Other Drug Use and Related Problems

In CHDS age 16 drinking latent class was not associated with age 21 cannabis and other illicit drug dependence after control for confounding [60]. No effects of age 15–16 drinking lifetime prevalence on other drug use at 24–25 were observed in the NYLS [33]. Despite this cohort being particularly closely associated with the “gateway” perspective (any prior use of one drug increasing risk of subsequent use of another), only cumulative use measured over the entire intervening period was associated with other drug use, thus “adolescent use retains no direct unique effect once use between adolescence and young adulthood is taken into account” [33]. In the SCS after 26–27 y of follow-up, a summary measure of problematic drinking among those who had not used cannabis at conscription was not associated with hospitalization or mortality with a drug use diagnosis (OR = 1.83 [0.97–3.45]) [18]. Among problem drinkers who had used cannabis, however, elevated risks of such drug problems were found (ever used ≤10 times OR = 5.60 [2.92–10.75], >10 times 3.34 [1.60–6.98]).

### Educational Attainment

No effects of adolescent drinking on any educational outcomes (school qualification, university enrolment, or degree) at age 21 remained after adjustment for confounding in CHDS [60]. In the NYLS there was no effect of having drunk alcohol ten times or more by age 15–16 on number of years in education by 24–25 y [33]. In the British NCDS, however, an effect of heavier past week drinking (>4 units male, >3 units female) at age 16 on subsequent educational attainment by age 42 was found among men only, using propensity scores to deal with confounding [54]. This effect was greater in working class men, where heavy drinkers were approximately 25% less likely to complete a degree than nonheavy drinkers. The difference was 10% among middle class men [54].

### Other Possible Consequences

The CHDS found no effect of age 16 drinking latent class on months unemployed, sexually transmitted infections, or pregnancy by age 21 [60]. Effects on numbers of sexual partners were identified to be robust to adjustment for background variables. Hospitalisation during 15 y of follow-up for any accidents, or gastro-intestinal, respiratory, musculo-skeletal, or infectious disorders in Stockholm County in the SCS was not significantly higher (OR 1.2 [0.9–1.6]) for heavier drinkers as compared to moderate drinkers as defined above [13]. The NYLS found no direct effects on a wide range of possible consequences of having ever drank alcohol ten times or more by age 15–16. Given the limitations of this measure it is perhaps not surprising that alcohol consequences, compared to other substances, were characterised as “benign” [33]. Finally, there were no effects of drinking frequency at age 18 on life satisfaction at age 29 in a western US school cohort [10], again in contrast to other substances.

### Additional Evidence from Other Studies

Evidence from studies not assessed as having stronger capacity for inference broadly agreed with the findings presented above. There was consistent evidence of effects on subsequent alcohol consumption and related problems. There were similarly mixed findings on possible mental health, tobacco smoking, and educational consequences. There are two principal exceptions: Although no effects on pregnancy among young women were found in CHDS, contrary findings emerged from two other cohorts [46],[59], one of which was restricted to pregnancy outcome by age 18 [59]. Also in three of the four studies that investigated possible effects on adult drug use or related problems, associations with at least one outcome measure in this area were identified [40],[46],[59]. In both cases, the vulnerability of these findings to bias and/or confounding should be remembered.

## Discussion

This systematic review has investigated whether late adolescent alcohol consumption is a time-limited activity without significant longer term consequences or whether it impacts upon adult health and well being. It is clear that the evidence base on long-term consequences is not as extensive nor as compelling as it could be. There are sparse data of sufficient quality to warrant making causal inferences on the broader health and social consequences of late adolescent drinking on the basis of the data evaluated here.

There is evidence from a single population-based cohort that late adolescent drinking can cause early death among men, principally through car crashes and suicides [12],[15]. There is a large evidence base attesting to the ongoing impacts of late adolescent drinking on adult drinking behaviours, though most studies cannot strongly support causal inferences because of their designs. There is robust evidence from one national cohort that apparent effects on later alcohol consumption persist beyond the age of 30, which is longer than had previously been understood [43]. Possible effects on subsequent alcohol problems including dependence are somewhat more complex than effects upon subsequent alcohol consumption per se. Evidence from multiple well-designed cohort studies indicates that other factors indicative of heightened psychosocial risk more broadly are also implicated. It is nonetheless striking that effects on alcohol problems assessed in the mid 30s appear to have been produced by elevated consumption in late adolescence in both SCS and MFCS, and to earlier ages in other studies. Findings from a rigorous birth cohort study on nonalcohol outcomes, however, demonstrate that many apparent effects of late adolescent drinking may be due to uncontrolled confounding [60]. Certainty about the long-term consequences of late adolescent drinking is thus not easily achieved.

Caution is also required because of the limitations of the present study. Our approach to the investigation of confounding in individual studies was necessarily constrained by weaknesses of the literature as a whole and by the comprehensive nature of our appraisal of possible consequences. For example, there are few studies that address family influences, both siblings and parents. Similarly, there was only a single study included that investigated genetic inheritance [58]. Confounding also needs to be considered in relation to more specific outcomes. For example, a CHDS study not included here by virtue of not having a drinking behavioural measure, found that the contribution of drink driving to traffic accidents is much reduced when other risky driver behaviours are taken into account [63]. Unless otherwise indicated in the Results section, traditional methods of investigation of confounding have been used in the studies covered and these can be criticised for their adequacy in dealing with residual confounding. Being designated as having stronger capacity for casual inference here should not be mistaken to indicate support for causal inference in relation to observed associations, nor that we view the treatment of psychosocial factors to have eliminated residual confounding. Conversely, not being so designated does not imply that a study is weak, rather, simply that it does not contribute as much as others to the aims of this review.

Bias was considered in relation to sample size and study attrition in our appraisal of study quality. Whilst this latter variable is long established as important to the evaluation of cohort studies, thinking about the former has been more recently advanced, and thus deserves elaboration and consideration of the impact on review findings. Small study effects have been recently observed within meta-analyses of trials [64]. These effects are related to publication bias (see below) though are more fundamentally due to the greater likelihood of bias in effect estimates in small studies compared to large studies [65]. We are not aware that the influence of small study effects has previously been considered within systematic reviews of cohort studies despite their potential for bias. At the outset, we judged that it was important to consider this possibility, and to use a simple means of so doing, given the challenges involved in summarising a large number of observational studies with substantial problems of bias and confounding. We took the decision to give additional weight to larger studies after piloting and before the main study data collection and analysis. The particular threshold we chose may be somewhat arbitrary, though it is not clear that moving this threshold upwards or downwards by a few hundred study participants would substantially influence our findings.

Two other important forms of bias were not directly involved in the determination of study quality. Almost all adolescent behavioural data were self-reported. These data are most likely to involve underestimation of true levels of drinking and its consequences for reasons of social desirability, though the possibility of exaggeration should also not be ignored [66],[67]. Self-report bias leads to underestimation of the true extent of the relationships between adolescent exposure and adult consequences, as would also be true if reporting error was random rather than systematic. This problem is compounded by the fact that the vast majority of the adult outcome data are also self-reported, making probable further underestimation of the true effects [68], notwithstanding the effects of repeated measures within cohorts. The SCS studies are a noteworthy exception to the reliance on self-reported outcomes. It would have been possible to have selected data reliability as a criterion for bias evaluation; this would not have meaningfully changed the results beyond giving greater prominence to SCS data.

Publication bias exerts influence in the opposite direction [69],[70]. If the studies reviewed here represent a biased sample of all the relevant studies that have been undertaken, then overestimation of actual effects occurs, which seems highly likely given the paucity of negative findings for alcohol outcomes. This threat to valid inference perhaps has its origins in the context of preliminary explorations of cohort study datasets. In these situations, if drinking is not found to be associated with outcomes of interest then the analyses may not be pursued. We may also have missed studies that meet inclusion criteria by virtue of their publication characteristics. This risk is inherent in the nature of this exercise and is heightened given the breadth of the outcomes investigated here. It seems likely, therefore, that the possibility of not having successfully identified all relevant studies is greater for nonalcohol compared to alcohol outcomes. At the outset we expected publication bias to be a greater threat to the validity of inferences made than small study effects. By its nature, however, we were not in a position to attribute this risk to individual included studies. Both publication and reporting bias pose profound threats to valid inference in this review whose magnitude is difficult to appreciate quantitatively.

Studies from a range of different national and cultural contexts have been included here and identified as providing a stronger basis for causal attribution, though these are entirely restricted to Anglophone and Northern European countries. There are no included studies from low- and middle-income countries, nor from any country with a Mediterranean drinking culture. Previous meta-analytic study in this area has identified national context to be particularly important to findings on alcohol consumption from cohort studies [3]. These studies also cover limited historical periods. Period effects were investigated across the different cohorts in the MFCS where only limited differences were identified [43].

Because there have been no previous systematic reviews of this literature, the research question addressed here is unusually broad. This approach led us to specify an end date for formal inclusion in the review, and inevitably further studies have since been published. For example, Huurre and colleagues [71] robustly identified continuities between heavy drinking at age 16 and hazardous drinking at age 32 in a Finnish study. A later MFCS report demonstrates the application of multilevel analyses to examine more advanced research questions on mediators and moderators of effects [72]. Other more recent studies that may have been included [73],[74] do not substantially change the picture obtained, though there will be other studies of which we are unaware and we expect this literature to continue to grow rapidly.

Notwithstanding the limitations of the evidence base and of this review, and attenuations over time in the strength of the direct effects, late adolescent alcohol consumption appears a probable cause of increased drinking well into adulthood, through to ages at which adult social roles have been achieved. Heavier drinking seems most likely, however, to be only one component in a complex causal process, whose contribution has probably been overestimated in previous studies because of uncontrolled confounding, setting aside the uncertainties induced by self-reported data. The importance of these data is highlighted in the context of work showing strong stability of drinking patterns through the fourth and fifth decades of life [3],[31],[75]. A wide range of health and other harms, such as liver cirrhosis, are caused by alcohol at middle and older ages [76],[77]. Late adolescent drinking, by virtue of its probable effect on long-term adult alcohol consumption is likely to contribute to the burden of alcohol-related disease. Continuities from adolescence to adulthood in drinking patterns have been observed across a range of measures including frequency of consumption and heavy drinking.

In this study it seems that alcohol consumption confers additional risk of alcohol problems both on those who are already more vulnerable in various ways to poorer health and psychosocial outcomes, and strikingly also among those who are not otherwise vulnerable. Possible effects on adult alcohol problems and dependence including hospitalisation identified here result from heavier drinking in adolescence without necessarily involving problems at younger ages. If these effects are confirmed, there are two important implications: (1) Reducing late adolescent alcohol consumption in the general population may be expected to make a long-term contribution to reducing the incidence of adult alcohol problems; (2) In more vulnerable populations, late adolescent drinking may be one cause among many of later difficulties, and its effects may be more severe and long-lasting [78]. Having relatively secure psychosocial resources may somewhat buffer these risks, and their consequent potential for adverse effects, but it does not remove them. These statements should be read with some caution given studies of mediators and moderators of these effects are lacking, limiting our understanding of their nature. Nevertheless, this systematic review affords more secure inference of the likely existence of these effects than has been possible previously. It is possible that relationships with alcohol forged during late adolescence may have cumulative lifetime drinking related consequences that are also simply not well captured by the existing literature.

The lack of convincing evidence of effects on nonalcohol outcomes is the product of an absence of evidence rather than strong evidence indicating no effects. A priori, one might expect that effects on nonalcohol outcomes would be weaker simply because they are less direct. To this extent, any such effects would be less durable and may be more likely to occur in high risk subgroups. There is also the possibility of reverse causation in relation to many of these consequences as the initiation of drinking in adolescence may have been preceded by many of the nonalcohol outcomes considered here. Only careful studies of adolescence may address this possibility.

There is also a clear need for high quality long-term prospective cohort studies in order to better understand the public health burden that is consequent on late adolescent drinking, both in relation to adult drinking and more broadly. A number of the cohorts included here were originally formed for prevention trials, and obtained some short-term evidence of benefit (for example the RAND and Seattle cohorts in Table 1). There is currently, however, an absence of experimental evidence of successful intervention modifying drinking during the late adolescent years leading to improved adult outcomes [79]. Long-term investment in rectifying this state of affairs should be a public health priority.

In addition to making both alcohol and heavy drinking less available, less acceptable, and more expensive [80],[81], these findings indicate a need for policy makers to encourage young people to be more cognisant of the long-term risks to adult health and well-being, and to act on this awareness in their decision making about whether and how much to drink [82]. This encouragement requires much more than the provision of accurate information about risks if it is to have any real prospect of influencing actual behaviour. Alcohol harm reduction has largely been concerned with reducing various risks inherent in drinking situations and their immediate aftermaths [81]. This study demonstrates the need to develop a longer term perspective on harm reduction.

## Supporting Information

Text S1 PRISMA Checklist.
(0.07 MB DOC)

## Abbreviations

AHRS: Adolescent Health Risk Study
CHDS: Christchurch Health and Development Study
DSM-IV: *Diagnostic and Statistical Manual of Mental Disorders, Fourth Edition*
NCDS: National Child Development Study
MFCS: Monitoring the Future Cohort Study
NYLS: New York Longitudinal Study
OR: odds ratio
SCS: Swedish Conscript Study
